# Selected Serum Biomarkers (Leptin, Chromogranin A, CA19-9, CEA) in Patients with Pancreatic Neuroendocrine Neoplasm and Associations with Metabolic Syndrome

**DOI:** 10.3390/cancers15082348

**Published:** 2023-04-18

**Authors:** Violetta Rosiek, Agnes Bocian-Jastrzębska, Beata Kos-Kudła

**Affiliations:** Department of Endocrinology and Neuroendocrine Tumors, Department of Pathophysiology and Endocrinology, Medical University of Silesia, 40-514 Katowice, Poland

**Keywords:** pancreatic neuroendocrine neoplasm, leptin, chromogranin A, CEA, CA19-9, metabolic syndrome

## Abstract

**Simple Summary:**

Pancreatic neuroendocrine neoplasms (PanNENs) represent approximately 30% of all gastro-entero-pancreatic NENs. According to current knowledge, the association between PanNENs and metabolic syndrome (MS) is not known. The study aimed to assess the relationships between selected serum biomarkers and metabolic syndrome (MS). We identified PanNEN patients with MS as having higher CEA levels, while those with a higher BMI and female sex showed higher leptin levels. This might reflect the relevant function of CEA and leptin in the metabolic abnormality diagnostics of PanNENs and deserves further evaluation to increase knowledge about the metabolic syndrome in PanNENs.

**Abstract:**

Metabolic abnormalities are well-known risk factors for many cancers, even though no clearly established link with pancreatic neuroendocrine neoplasms (PanNENs) has yet been investigated. This research aimed to assess the serum levels of leptin, chromogranin A (CgA), carbohydrate antigen 19-9 (CA19-9), and carcinoembryonic antigen (CEA) in patients with PanNENs and to search for associations between PanNENs, these selected serum biomarkers, and metabolic abnormalities in the form of metabolic syndrome (MS). Second, we aimed to investigate whether MS increases the risk of PanNENs. The serum concentrations of biomarkers, metabolic parameters (glucose, cholesterol, triglycerides), and anthropometric measurements (weight, height, BMI) were assessed in 106 patients with PanNENs and 40 healthy volunteers. Patients with PanNENs showed higher serum concentrations of CA19-9, CEA, and CgA in comparison to controls (*p* < 0.001, *p* = 0.042, and *p* = 0.025, respectively). Statistically significant differences in CEA levels were found in PanNENs patients with MS (*p* = 0.043). PanNENs patients with a BMI ≥ 25 kg/m^2^ and who were female exhibited significantly higher leptin levels (*p* < 0.001 and *p* = 0.013, respectively). Additionally, this study reflects the importance of determining markers. Future research should focus on understanding the impact of metabolic disturbances on PanNENs and accounting for the relationship between PanNENs and MS, such as other malignancies.

## 1. Introduction

Pancreatic neuroendocrine neoplasms (PanNENs) represent 4% of all neuroendocrine neoplasms (NENs) that develop from cells of the diffuse endocrine system [1]. The incidence of PanNENs, both functional and non-functional, has constantly increased over the last few years and amounts to approximately 8–10 million cases per year [2,3,4]. Due to the lack of characteristic symptoms of hormonal activity, there are asymptomatic or manifest symptoms such as abdominal pain, nausea, vomiting, and weight loss when they reach a large size, causing signs of local invasiveness [1,5]. The treatment of PanNENs is highly dependent on the clinical stage, tumor grade, and differentiation.

Metabolic syndrome (MS) is not a separate disease but a group of conditions, such as obesity, hypertension, hyperglycemia, and dyslipidemia. There are many classifications of MS (see Table S1 in the supplementary materials). According to the latest update (2021) [6] on the pathophysiology and management of metabolic syndrome, it represents a group of metabolic abnormalities, including abdominal obesity, impaired insulin sensitivity, high blood pressure, and atherogenic dyslipidemia. Both genetic and acquired factors are involved in the pathogenesis of MS, leading to insulin resistance and chronic inflammation and significantly increasing the risk of developing diabetes and cardiovascular disease [7].

Currently, we know that adipose tissue is part of the endocrine system as a result of producing adipokines, which act similarly to hormones and can also promote cancerogenesis and increase tumor progression. The most well-known adipocyte-released peptide hormone is leptin. It has been proven that leptin functions similarly to a neoplastic process supporter in cancer [8]. Leptin overexpression has been described in multiple types of cancer, such as esophageal cancer, hepatocellular carcinoma, gallbladder cancer, pancreatic cancer, colorectal cancer, renal cell carcinoma, prostate cancer, bladder cancer, breast cancer, endometrial cancer, ovarian cancer, and papillary thyroid cancer [9,10,11]. However, the role of leptin in PanNENs has not been thoroughly evaluated and understood yet. Serum leptin concentration meaningfully correlates with fat mass and is substantially elevated in patients suffering from obesity, which leads to metabolic diseases being elements of MS.

The neoplastic markers, determined mainly in pancreatic cancer, are carbohydrate antigen 19-9 (CA19-9) and carcinoembryonic antigen (CEA) (less often). They are elevated in 75–85% of patients but may also indicate cancer of other organs and inflammatory processes. CA19-9 is a cell-surface glycoprotein complex and is produced by human pancreatic, biliary ductal, gastric, and colon cells. The level of CA19-9 can be elevated in several benign gastrointestinal disorders, but the plasmatic concentration is severely increased in pancreatic, biliary, and gastrointestinal cancers [12]. Carcinoembryonic antigen (CEA), a non-specific serum biomarker, functions as a prognostic biomarker and can be used in monitoring the treatment of many malignancies, such as gastric, pancreatic, and colorectal cancers. The constant increase in CEA levels is usually in connection with the progression of the disease. Furthermore, testing CEA levels is valuable for detecting local or distant recurrence. In most of the researchers’ opinions, CA19-9 sensitivity is much inferior to that of CEA, and elevated CA19-9 levels are a poor prognostic factor [13].

Chromogranin A (CgA) is a non-specific marker of all NENs and PanNENs and can only indicate the PanNENs’ presence and may help with follow-up of the disease course [14]. It also works as a prognostic factor for survival and a marker for the treatment of NENs (an increased CgA level may reflect a lack of response to therapy). An early decrease in CgA level is also a favorable prognostic factor for progression-free survival. One of CgA’s bioactive peptide fragments is 21-amino-acid-long catestatin, which acts in an opposite way to insulin, inhibiting lipogenesis and increasing lipolysis in adipose tissue by inhibiting the α2-adrenergic receptor, enhancing leptin signaling [15,16]. This shows an association between CgA, leptin, and metabolic homeostasis. The relationship between diabetes, obesity, and pancreatic cancer was described [17], but between PanNENs and metabolic abnormalities is not fully known. Could leptin and other biomarkers mediate these relationships? We set as our main goal finding associations between metabolic abnormalities and serum levels of leptin, CgA, CA19-9, and CEA in patients with PanNENs. An additional aim of our work was to investigate whether metabolic abnormalities, in the form of metabolic syndrome, increased the risk of PanNENs.

## 2. Materials and Methods

### 2.1. Study Participants

The study group consisted of 106 patients with PanNENs and 40 healthy subjects. All PanNENs patients were recruited from those admitted to the Department of Endocrinology and Neuroendocrine Tumors, the European Neuroendocrine Tumor Society Center of Excellence, and the Medical University of Silesia, Poland. As the control group, the healthy subjects were recruited from the hospital and outpatient clinic personnel. For all patients with PanNEN, a pathological diagnosis according to the WHO’s 2019 classification [18] and the American Joint Committee on Cancer/Union for International Cancer Control’s 2017 type [19] was confirmed, and written informed consent to participate in the study was obtained. The subjects were excluded if they presented the following exclusion criteria: lack of informed consent to participate in the study; other cancer or a history of malignancy; kidney or hepatic failure; age less than 18; pregnancy; and lactation.

The serum concentrations of selected biomarkers (leptin, CgA, CA19-9, and CEA), metabolic parameters (glucose, cholesterol, triglycerides), and anthropometric measurements (weight, height, BMI) in the study groups were assessed.

In our study group, we recognized MS according to modified criteria as having obesity (BMI 30 kg/m^2^) and at least two of the following:

1 Hypertriglyceridemia (≥150 mg/dL) or treatment of hypertriglyceridemia;
2 Low HDL cholesterol levels (<40 mg/dL in men or <50 mg/dL in women) or hypercholesterolemia drug administration;
3 Elevated fasting glucose (≥100 mg/dL) or treatment of diabetes mellitus;
4 Hypertension (≥130/85 mmHg) or treatment of hypertension.

The levels of CgA, glucose, total cholesterol, and triglycerides, as well as information on age, sex, weight, height, and BMI of the PanNEN patients, were established through the patients’ hospital records.

Various factors cause a substantial increase in the CgA concentration, such as receiving proton pump inhibitors and histamine H2-receptor blockers, atrophic gastritis, renal failure, chronic inflammations, non-neoplastic diseases (gastrointestinal, cardiovascular, and endocrine), and other neoplasms [15]. Therefore, excluding them during the serum CgA level measurement of study participants was obligatory.

PanNEN patients were categorized into the following subgroups:

1 Normal BMI (BMI 20–24.9) vs. higher BMI (BMI ≥ 25);
2 With MS vs. without MS;
3 Gender (male vs. female).

### 2.2. Assessment of Serum Biomarkers

Venous blood samples were taken from all study participants. After centrifugation, the serum collection was stored at −80 °C until analysis. After that, SBM containing leptin, CA19-9, and CEA were evaluated with commercially available sets: leptin ELISA kits, CA19-9, and CEA with EIA kits. The reference ranges for serum biomarkers were as follows: for leptin, 0.4–15.98 ng/mL (men) and 3.47–58.63 ng/mL (women); for CA19-9, 0–29 U/mL; and for CEA, 0.5–9.1 µg/L.

Leptin metrics: The method’s sensitivity was 0.2 ng/mL; intra-assay precision and inter-assay precision were 4.2–7.6% and 4.4–6.7%, respectively.

CA19-9 metrics: The sensitivity of the method was <1 U/mL; intra-assay precision and inter-assay precision were 3.3–4.5% and 6.2–7.0%, respectively.

CEA metrics: The sensitivity of the method was <0.25 µg/L; intra-assay precision and inter-assay precision were 2.1–2.7% and 1.5–2.7%, respectively.

### 2.3. Statistical Analysis

The Shapiro–Wilk normality test identified that all biomarker values were not normally distributed. The comparisons of all analyzed parameters between subgroups (PanNENs vs. controls, normal BMI vs. higher BMI, with MS vs. without MS, and male vs. female) were made using the nonparametric Mann–Whitney U test. AUC was calculated as the area under the receiver-operating characteristic (ROC) curve. We used logistic regression to undertake the differentiation between the two patient groups. In the first step, we built a predictive model to differentiate patients with PanNENs from controls; next, we tried to assess the capacity for differentiating between PanNEN patients with normal and high BMI depending on the presence/absence of MS, and finally sex (females from males). The correlation coefficient between biochemical, metabolic, and anthropometric parameters and BMI was calculated using Spearman’s rank correlation coefficient. The results for groups and subgroups are presented as the mean, standard deviation, and median. Statistical significance was assumed at a value of ≤0.01.

Statistical analyses were performed using Statistics v. 13.0 (StatSoft, Kraków, Poland) software.

## 3. Results

### 3.1. Pancreatic Neuroendocrine Neoplasm Patients vs. Controls

One hundred and six patients with PanNEN and forty controls were enrolled in the study. The mean age of the patients with PanNEN was 52.59 [years], and in the control group, it was 48.53 [years]. The patients with PanNEN were grouped according to BMI [kg/m^2^], MS presence, and sex. In total, 54 patients (51%) were of normal weight (BMI 20–24.9 kg/m^2^), and 52 patients (49%) had a BMI ≥ 25 kg/m^2^. MS was confirmed in 27 (25%) patients with PanNEN. The demographic, biochemical, and clinical characteristics of the 146 study participants are presented in Table 1.

**Figure 1:**
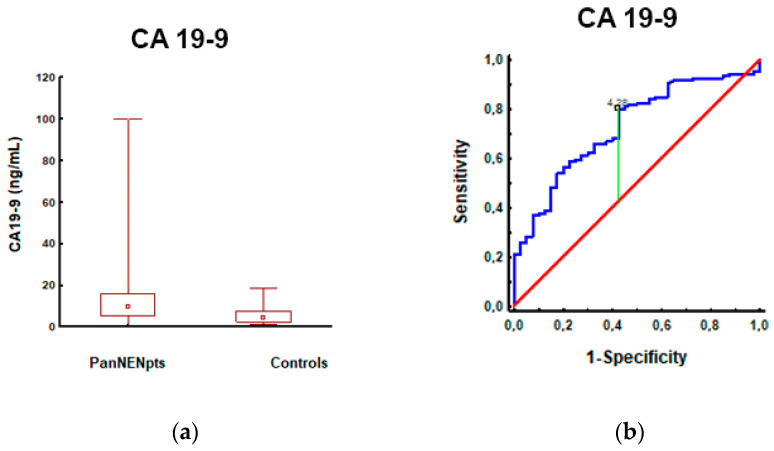
CA19-9 levels in patients with PanNEN and controls: (**a**) CA19-9 measurements were significantly higher in patients with PanNEN (12.89 ± 14.66; 9.43; *n* = 106) compared to controls (5.60 ± 4.46; 4.10; *n* = 63) (*p* < 0.001); (**b**) The AUROC for CA19-9 levels in patients with PanNEN and controls: the AUROC (red line) for differentiating patients with PanNEN from controls was 0.73 (95%CI: 0.64–0.82; (*p* < 0.001). A maximum AUC = 1 identifies an ideal (perfect) differentiation between disease and non-disease subjects. The diagonal line (AUC = 0.5) corresponds to chance discrimination. The CA19-9 AUC > 0.7 (red line) indicates that it is a fair biomarker for patients with PanNEN.

The serum biomarker that results in PanNENs was compared to controls, and in the PanNEN subgroup, it was reached depending on MS presence, high BMI, and gender. Significantly higher concentrations of CA19-9 (Figure 1), CgA, and CEA were observed in PanNEN patients compared to controls (*p* < 0.05). Leptin levels were similar in both groups (Table S2 in Supplementary Materials).

In addition, an AUC analysis of CEA and CgA could differentiate PanNENs from controls; although significant, it should be noted that an AUC of <0.7 would be considered a poor biomarker (Table S3 in the supplementary materials). CA19-9 had the highest AUC (0.73, *p* < 0.001, sensitivity/specificity of 80/58%, respectively), differentiating PanNENs from controls, and seems to have the diagnostic capacity of a fair single biomarker (Figure 1).

### 3.2. Patients with Pancreatic Neuroendocrine Tumors with Normal BMI vs. High BMI

In the second step of our study, we divided the PanNEN subjects into two groups: those with normal BMI (2024.9 kg/m^2^) and those with higher BMI (≥25 kg/m^2^). Table 2 shows the characteristics of 51 patients with normal BMI and 55 patients with higher BMI.

**Figure 2:**
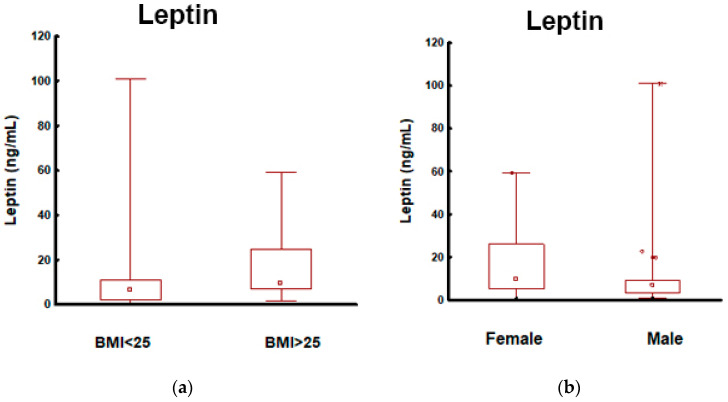

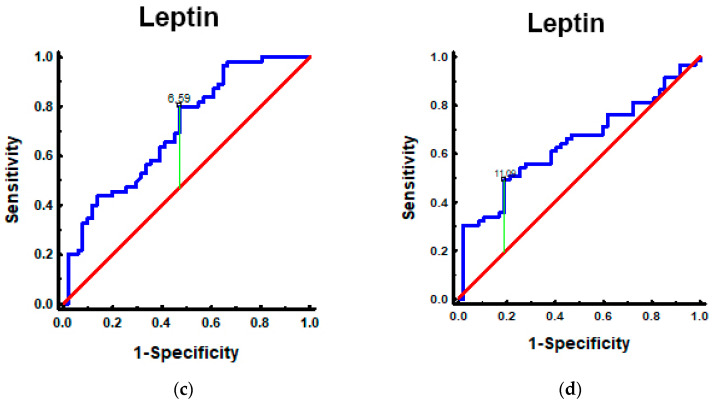
Leptin in patients with PanNEN according to BMI and sex: (**a**) Mann–Whitney test. Leptin in PanNEN patients with normal BMI and elevated BMI: measurements of the leptin identified elevated levels in patients with PanNEN with BMI ≥ 25 kg/m^2^ vs. BMI < 25 kg/m^2^ (*p* < 0.001); (**b**) Mann–Whitney test. Leptin in patients with PanNEN according to the sex: measurements of the leptin identified elevated levels in patients with PanNEN: female vs. male (*p* = 0.013); (**c**) the AUROC for leptin levels in patients with PanNEN with BMI ≥ 25 kg/m^2^ and BMI < 25 kg/m^2^. The AUROC (blue line) for differentiating patients with PanNEN with BMI ≥ 25 kg/m^2^ from BMI < 25 kg/m^2^ was 0.71 (95%CI: 0.61–0.80. *p* < 0.001); (**d**) the AUROC for leptin levels in patients with PanNEN: female and male. The AUROC (blue line) for differentiating females and males was 0.64 (95%CI: 0.54–0.75. *p* = 0.008).

Compared with patients with BMI < 25 kg/m^2^ versus those with BMI ≥ 25 kg/m^2^, there were no significant differences in CA19-9, CEA, and CgA levels. No patient had jaundice at the time of blood sampling; therefore, CA19-9 was not falsely elevated in this group. We observed significantly higher leptin concentrations in PanNEN patients with a higher BMI (Figure 2).

The metrics of selected biomarkers (leptin, CA19-9, CEA, and CgA) in the differentiation of PanNENs depending on BMI are presented in Table S4 of the Supplementary Materials. Among them, only leptin had the capacity for differentiating between PanNEN patients with normal and high BMI (AUC 0.71; *p* < 0.001; sensitivity 80%; specificity of 53% indicating that it is a fair biomarker for PanNENs with elevated BMI) (Figure 2).

### 3.3. Patients with Pancreatic Neuroendocrine Neoplasms (PanNENs) with Metabolic Syndrome (MS) vs. PanNENs without MS

MS was diagnosed in 27 (25%) patients with PanNEN. Table S5 in the supplementary materials presents a comparison of anthropometric and biochemical parameters depending on the presence/absence of MS. Except for CEA levels, no statistically significant differences were found in other serum marker levels in patients with PanNEN and MS.

The mean CEA concentration was 2.07 ± 1.81 µg/L in the patients and PanNEN with MS and 1.50 ± 1.50 µg/L in those without MS, and the difference was statistically significant (*p* = 0.043).

The metrics of biomarkers (AUC, sensitivity, specificity, and accuracy) used in the differentiation of PanNENs depending on the presence/absence of MS showed only CEA to be a significant difference in both subgroups (AUC 0.63, *p* = 0.036, and sensitivity/specificity of 56/75%, respectively). It indicates a poor biomarker (Table S6 in the supplementary materials).

### 3.4. Patients with Pancreatic Neuroendocrine Tumors: Male vs. Female

Sex was included in the analysis as an additional factor differentiating PanNEN patients. Comparing anthropometric and biochemical parameters in the PanNEN patients depending on the sex showed that females tended to have a higher leptin level than males (*p* = 0.013) (Figure 2b). The sex of the PanNEN patients did not affect the other serum biomarker concentrations such as CA19-9, CEA, and CgA (Table S7 of the supplementary materials).

The leptin AUC analysis indicates that it could differentiate females from males in patients with PanNEN (Table S8 of supplementary materials), but AUC = 0.64 (blue line) means that it is, unfortunately, a poor single biomarker for PanNEN females (Figure 2).

### 3.5. Spearman’s Correlation

In the last step of the study, we assessed the correlations between BMI and analyzed variables in all PanNEN subjects. We observed significant associations with age, weight, cholesterol, TGs, glucose, and leptin levels (Table 3). However, no associations were found with CA19-9, CEA, or CgA serum concentrations.

## 4. Discussion

In epidemiological studies, MS occurrence varies between 20% and 50% of the population [20], and the prevalence of MS increases even more as BMI increases. The incidence of neoplasms (also known as NENs) has been growing over the last few decades with the increasing incidence of MS. It is not yet apparent whether the MS compounds could be associated with PanNENs or could influence their prognosis and survival. In this study, we aimed to provide data, from Italian researchers, on the association of PanNENs with the metabolic disturbances typical of MS [21]. Obesity causes a variety of systemic alternations, including changed levels of adipokines, hormones, and cytokines, and is associated with oxidative stress and dysbiosis. Those abnormalities increase the risk of developing cancer [22,23]. Furthermore, obesity is associated with chronic systemic inflammation, which has been suggested to play a role in cancerogenesis and the gastrointestinal tract [24]. Many researchers proposed the association between cancer and metabolic disorders several years ago [25]. Weight loss and a healthy diet undeniably decrease cancer risk, and antidiabetic medications such as metformin may have anticancer effects independent of their hypoglycemic effects [26].

Pancreatic cancer is an obesity- and diabetes-related cancer [17,27,28,29]. These two components of MS promote cancerogenesis in many ways [17]. However, the metabolic risk factors and neoplastic mechanisms in pancreatic NENs are still unknown. According to a systematic review and meta-analysis by Leoncini et al., the most relevant risk factor for NEN all-sites development (also known as PanNENs) is a high BMI, followed by diabetes [30]. Some studies regarding the relation between PanNENs and BMI found opposite results: two case-control studies have shown that a high BMI increases PanNEN development risk [31,32]. The findings of the Israeli researchers suggest different associations of risk factors with individual GEP-NENS: increased height and weight do not increase the risk of PanNENs, but gastric NENs do [33]. Different results were obtained by Italian researchers from referral centers for NENs. Their results confirm that obesity and DM2 are independent risk factors for PanNENs. DM2 was also associated with more advanced and progressive diseases. The authors suggest metformin’s role as a protective factor in DM2 subjects [34]. The urgent need for more detailed research on the risk factors of individual NENs should be noted. Recent data also suggest a potential link between DM2 and insulinoma. In a minireview, Duville et al. discuss the cellular and molecular mechanisms that may explain these interactions: some specific gene mutations contribute to both DM2 and insulinoma, reinforcing the association between these diseases. In contrast, other modifications have opposing effects on DM2 and insulinoma [31]. In our study group of PanNEN patients with BMI ≥ 25 kg/m^2^, we noted higher levels of cholesterol, triglycerides, glucose, and older age than patients with BMI < 25 kg/m^2^. Thus, these are the consequences of metabolic complications associated with being overweight and obese.

Does a correlation between NENs and metabolic disturbances occur more often? Santos et al. [35] have shown the association between MS components and well-differentiated (WD) gastro-entero-pancreatic NENs (GEP-NENs): WD GEP-NENs were related to elevated waist circumference, fasting plasma glucose, triglycerides, and severe insulin resistance. In 2019, Santos et al. [36] evaluated whether the presence of MS or any MS individual component affects GEP-NEN features at the time of diagnosis. They found that patients with GEP-NEN and MS had a higher risk of presenting with a lower tumor grade and disseminated disease [36]. Their results need to be confirmed with larger-scale studies, but they tentatively provide a connection between MS and NENs. In our study, MS was diagnosed in 27 of the 106 patients with PanNENs, and we also observed elevated fasting plasma glucose and triglyceride concentrations in this group. We did not investigate the serum biomarkers levels regarding PanNENs grades and stages, but we plan to design such a study.

If possible, surgical treatment of WD pancreatic neuroendocrine neoplasm G1/G2 (PanNEN G1/PanNEN G2) is the treatment of choice [1]. Although surgical procedures are the treatment of choice to increase survival in these patients, they are not without complications, such as postoperative fistula. Oweira H et al. widely discussed the associations between a postoperative pancreatic fistula risk and metabolic syndrome, as well as high BMI after distal pancreatectomy [37]. Therefore, the decision regarding the PanNEN treatment method should be made by physicians experienced in the treatment of neuroendocrine neoplasms, given various risk factors (including a high body mass index) and comorbidities.

Leptin, as an adipocyte-secreted hormone, has markedly higher serum concentrations in persons with a high BMI and women than in men (because body fat percentages are higher in women than in men) [38]. These data are confirmed by our results: according to BMI and sex, we found significantly higher leptin levels in PanNEN patients with a BMI ≥ 25 kg/m^2^ and in the female sex (*p* < 0.001 and *p* = 0.013, respectively). Spearman’s coefficient also showed a significant positive correlation between BMI and leptin level (r = 0.49, *p* < 0.001). These findings confirmed that being overweight and obese, as well as female gender, were related to hyperleptinemia.

The plasma levels of acylated ghrelin, another hormone that regulates hunger in opposition to leptin, decreased in patients with insulinoma, probably due to hyperinsulinemia and obesity in the patients [39].

In addition to the amount, women have more subcutaneous fat relative to visceral fat than men [38]. Waist circumference is positively correlated with visceral adipose. Another parameter, the visceral adiposity index (VAI), has been suggested as a gender-specific indicator of adipose dysfunction and was used by Barrela et al. to investigate it as an early predictor of MS in patients with gastroenteropancreatic neuroendocrine neoplasms (GEP-NENs) [40]. The authors demonstrate that MS presence and visceral adiposity dysfunction, evaluated by VAI, were higher in G2 than in G1 patients (*p* < 0.001), in patients with progressive disease, and in metastatic patients. Moreover, a correlation with worse clinicopathological characteristics in GEP-NEN has been shown. The same authors performed exciting research on the role of chronotype in GEP-NEN patients, arguing that GEP-NEN patients, especially with the presence of metastasis (grading G2) and progressive disease, have an unhealthy metabolic profile that is more commonly associated with an evening chronotype. Importantly, these results provide a basis for including the assessment of chronotype as an adjunctive tool for preventing metabolic alterations and tumor aggressiveness in GEP-NEN [41].

Furthermore, leptin promotes cancerogenesis in many processes, including proliferation, cell survival, and angiogenesis, with consequent cancer progression. In the opinion of Stolzenberg-Solomon et al. (systematic reviews and meta- and pooled analyses), there is an association between higher prediagnostic circulating leptin concentrations and increased pancreatic cancer risk observed in patients with longer follow-ups. Reverse causation-reducing blood leptin concentrations explained the lack of a positive association in earlier years of follow-up due to weight loss in advanced disease [42]. Another study by Yuan shows significantly decreased circulating leptin levels in pancreatic cancer patients compared with non-pancreatic cancer individuals [43]. In our results, leptin concentrations were similar in both groups (PanNENs and controls). Although we did not indicate differences in leptin concentrations in PanNEN patients and management, due to the proven role of leptin in cancerogenesis and pancreatic cancer, further research in more numerous NEN cohorts is required. In our analysis, sex was an additional factor in differentiating PanNEN patients. We reveal that females tend to have a higher leptin concentration than males (*p* = 0.013).

We observed significantly higher concentrations of CA19-9, CEA, and CgA in PanNEN patients compared to controls. Still, in the AUC, differentiating PanNENs from management, CA19-9 had the highest rate (AUC 0.73, *p* < 0.001, sensitivity/specificity of 80/58%, respectively). It seems to have a diagnostic capacity as a fair single biomarker. CA19-9 is the neoplastic marker, determined mainly in pancreatic cancer. Unfortunately, it is not recognized as a typical marker for PanNENs. Considering the presence/absence of MS, PanNEN patients with MS had higher concentrations of CA19-9, CEA, and CgA. However, only the CEA concentrations (*p* = 0.043) compared to other SBMs were significantly different in these subgroups.

In the present study, the sex of the PanNEN patients did not affect the the serum biomarkers concentrations, such as CA19-9, CEA, and CgA. The literature shows that sex impacts many types of cancer prevalence, progression rates, therapeutic response, and cancer marker concentrations [44,45,46]. These differences could result from a more significant accumulation of subcutaneous fat relative to visceral fat in women than in men.

## 5. Conclusions

Patients with PanNENs showed higher serum concentrations of CA19-9, CEA, and CgA than healthy subjects. This study can reflect the relevant meaning of these biomarkers in PanNEN detection. In addition, the serum leptin concentration showed no statistical differences in both groups. Except for CEA concentrations, no statistically significant differences were found in other SBM concentrations in PanNEN patients with MS. According to BMI and sex, PanNEN patients with a BMI ≥ 25 kg/m^2^ and female sex exhibited significantly higher leptin concentrations. Furthermore, future research should focus on understanding the impact of obesity and metabolic disturbances on NENs and accounting for the relationship between NENs and MS, as with other malignancies.

## 6. Limitations of the Study

The subgroup analysis was only undertaken in the pancreatic neuroendocrine neoplasm (PanNEN) group because we did not have these metabolic data in the control group. This is indeed the major limitation of our study.

## Figures and Tables

**Table 1:** Demographic, clinical, metabolic, and laboratory characteristics of patients with pancreatic neuroendocrine neoplasm (PanNEN) and controls were included in the study.

Variables	PanNEN *n* = 106	Controls *n* = 40
Sex		
Women	59 (56%)	31 (77.5%)
Men	47 (44%)	9 (22.5%)
Age, years: mean (range)	52.59 (19–79)	48.53 (22–76)
Women	53.15 (21–79)	47.45 (22–76)
Men	52.02 (19–77)	52.22 (23–70)
Age [years]		
<55	54 (51%)	22 (55%)
≥55	52 (49%)	18 (45%)
Metabolic syndrome		N/A
No	79 (75%)	
Yes	27 (25%)	
BMI [kg/m^2^]		N/A
<25	51 (48%)	
≥25	55 (52%)	
Hyperglycemia		N/A
No	57 (54%)	
Yes	49 (46%)	
IFG	15 (14%)	
IGT	11 (10%)	
DM 2	23 (22%)	
Hyperlipidemia		N/A
Hypertriglyceridemia	20 (19%)	
Hypercholesterolemia	51 (48%)	
Hypertension		N/A
No	72 (68%)	
Yes	34 (32%)	

Data are shown as numbers and percentages (%). Abbreviations: BMI, body mass index; IFG, impaired fasting glucose; IGT, impaired glucose tolerance; DM 2, type 2 diabetes.

**Table 2:** Demographic, clinical, metabolic, and laboratory characteristics of patients with PanNENs depend on the body mass index (BMI) (Mann–Whitney U test).

	BMI < 25; *n* = 51 (mean ± SD; Median)	BMI ≥ 25; *n* = 55 (mean ± SD; Median)	*p*
Age (years)	49.57 ± 13.83; 52.40	55.51 ± 13.00; 57.77	NS
Weight (kg)	61.64 ± 10.32; 61.00	80.98 ± 11.63; 81.00	<0.001
Height (cm)	168.73 ± 10.29; 167.00	169.58 ± 8.53; 170.00	NS
BMI (kg/m^2^)	21.57 ± 2.37; 22.40	28.11 ± 3.18; 27.34	<0.001
Cholesterol (mg/dL)	183.61 ± 56.02; 178.00	194.62 ± 42.11; 192.00	NS
TGs (mg/dL)	100.84 ± 62.81; 82.00	116.40 ± 56.44; 98.00	NS
Glucose (mg/dL)	90.22 ± 17.84; 90.20	99.63 ± 26.93; 96.00	NS
Leptin (ng/mL)	9.21 ± 14.70; 6.33	16.03 ± 13.60; 9.36	<0.001
CA19-9 (U/mL)	10.67 ± 8.64; 7.87	14.96 ± 18.42; 11.09	NS
CEA (µg/L)	1.52 ± 1.28; 1.06	1.76 ± 1.85; 1.21	NS
CgA (ug/L)	266.44 ± 597.89; 52.90	87.10 ± 139.60; 42.19	NS

Abbreviations: BMI, body mass index; SD. standard deviation; TGs, triglycerides; CA19-9, carbohydrate antigen 19-9; CEA, carcinoembryonic antigen; CgA, chromogranin A; NS, not significant.

**Table 3:** Spearman’s correlations between BMI and clinical and biochemical characteristics in all pancreatic neuroendocrine neoplasm patients (presented only as statistically significant at *p* < 0.01).

Parameter	R Spearman	*p*
Age (years)	0.31	0.001
Weight (kg)	0.83	<0.001
Height (cm)	0.06	NS
Cholesterol (mg/dL)	0.21	NS
TGs (mg/dL)	0.26	<0.01
Glucose (mg/dL)	0.23	NS
Leptin (ng/mL)	0.49	<0.001
CA19-9 (U/mL)	0.03	NS
CEA (µg/L)	0.02	NS
CgA (ug/L)	−0.14	NS

Abbreviations: see Table 2.

## Data Availability

All data are available upon reasonable request.

## Supplementary Materials

The following supporting information can be downloaded at: https://www.mdpi.com/article/10.3390/cancers15082348/s1, Table S1: Diabetes Federation diagnostic criteria of metabolic syndrome: Table S2: The comparison of the studied circulating markers in patients with pancreatic neuroendocrine tumors and controls; Table S3: The selected serum markers metrics in the detection of pancreatic neuroendocrine tumors; Table S4: The selected serum markers metrics in the differentiation of pancreatic neuroendocrine tumors according to BMI; Table S5: The comparison of anthropometric and biochemical parameters depending on the presence/absence of the metabolic syndrome in patients with pancreatic neuroendocrine tumors; Table S6: The selected serum markers metrics in the differentiation of pancreatic neuroendocrine tumors depend on the presence/absence of the metabolic syndrome; Table S7: The comparison of anthropometric and biochemical parameters depending on the sex in patients with pancreatic neuroendocrine tumors; Table S8: The selected serum markers metrics in the differentiation of pancreatic neuroendocrine tumors depending on the sex.

## References

[1] Kos-Kudła B., Rosiek V., Borowska M., Bednarczuk T., Bolanowski M., Chmielik E., Ćwikła J.B., Foltyn W., Gisterek I., Handkiewicz-Junak D. (2022). Pancreatic neuroendocrine neoplasms—Update of the diagnostic and therapeutic guidelines (recommended by the Polish Network of Neuroendocrine Tumours). Endokrynol. Pol..

[2] Dasari A., Shen C., Halperin D., Zhao B., Zhou S., Xu Y., Shih T., Yao J. (2017). Trends in the incidence, prevalence, and survival outcomes in patients with neuroendocrine tumors in the United States. JAMA Oncol..

[3] Howe R., Merchant J., Conrad N., Keutgen C., Hallet X., Drebin J., Minter R.M., Lairmore T.C., Tseng J.F., Zeh H.J. (2020). The North American Neuroendocrine Tumor Society Consensus Paper on the Surgical Management of Pancreatic Neuroendocrine Tumors. Pancreas.

[4] Halfdanarson T.R., Strosberg J.R., Tang L., Bellizzi A.M., Bergsland E.K., O’Dorisio T.M., Halperin D.M., Fishbein L., Eads J., Hope T.A. (2020). The North American Neuroendocrine Tumor Society Consensus Guidelines for Surveillance and Medical Management of Pancreatic Neuroendocrine Tumors. Pancreas.

[5] Pavel M., Öberg K., Falconi M., Krenning E.P., Sundin A., Perren A., Berruti A. (2020). Gastroenteropancreatic neuroendocrine neoplasms: ESMO Clinical Practice Guidelines for diagnosis, treatment, and follow-up. Ann. Oncol..

[6] Fahed G., Aoun L., Bou-Zerdan M., Allam S., Bou-Zerdan M., Bouferraa Y., Assi H.I. (2022). Metabolic Syndrome: Updates on Pathophysiology and Management in 2021. Int. J. Mol. Sci..

[7] Alberti K.G.M.M., Zimmet P., Shaw J. (2006). Metabolic syndrome—A new world-wide definition. A consensus statement from the International Diabetes Federation. Diabetes Med..

[8] Ray A., Cleary M.P. (2017). The potential role of leptin in tumor invasion and metastasis. Cytokine Growth Factor Rev..

[9] Jiménez-Cortegana C., López-Saavedra A., Sánchez-Jiménez F., Pérez-Pérez A., Castiñeiras J., Virizuela-Echaburu J.A., de la Cruz-Merino L.D.L., Sánchez-Margalet V. (2021). Leptin, both bad and good actor in cancer. Biomolecules.

[10] Siemińska L., Borowski A., Marek B., Nowak M., Kajdaniuk D., Warakomski J., Kos-Kudła B. (2018). Serum concentrations of adipokines in men with prostate cancer and benign prostate hyperplasia. Endokrynol. Pol..

[11] Warakomski J., Romuk E., Jarząb B., Krajewska J., Siemińska L. (2018). Concentrations of selected adipokines, interleukin-6 and Vitamin D in patients with papillary thyroid carcinoma in respect to thyroid cancer stages. Int. J. Endocrinol..

[12] Lee T., Teng T.Z.J., Shelat V.G. (2020). Carbohydrate antigen 19-9—Tumor marker: Past, present, and future. World J. Gastrointest. Surg..

[13] Lech G., Słotwiński R., Słodkowski M., Krasnodębski I.W. (2016). Colorectal cancer tumour markers and biomarkers: Recent therapeutic advances. World J. Gastroenterol..

[14] Kos-Kudła B., Foltyn W., Malczewska A., Bednarczuk T., Bolanowski M., Borowska M., Chmielik E., Ćwikła J.B., Gisterek I., Handkiewicz-Junak D. (2022). Update of the diagnostic and therapeutic guidelines for gastro-entero-pancreatic neuroendocrine neoplasms (recommended by the Polish Network of Neuroendocrine Tumours). Endokrynol. Pol..

[15] Muntjewerff E.M., Dunkel G., Nicolasen M.J.T., Mahata S.K., Van Den Bogaart G. (2018). Catestatin as a target for treatment of inflammatory diseases. Front. Immunol..

[16] Bourebaba Y., Mularczyk M., Marycz K., Bourebaba L. (2021). Catestatin peptide of chromogranin A as a potential new target for several risk factors management in the course of metabolic syndrome. Biomed. Pharmacother..

[17] Paternoster S., Falasca M. (2020). The intricate relationship between diabetes, obesity and pancreatic cancer. Biochim. Biophys. Acta Rev. Cancer.

[18] Nagtegaal I.D., Odze R.D., Klimstra D., Paradis V., Rugge M., Schirmacher P., Washington K.M., Carneiro F., Cree I.A. (2020). The 2019 WHO classification of tumors of the digestive system. Histopathology.

[19] Bałdys-Waligórska A., Nowak A. (2021). Neuroendocrine neoplasms of the digestive system—Current classification and terminology. Nowotwory.

[20] Raposo L. (2021). Metabolic syndrome in Poland: The WOBASZ II study. Pol. Arch. Intern. Med..

[21] Natalicchio A., Faggiano A., Zatelli M.C., Argentiero A., D’Oronzo S., Marrano N., Beretta G.D., Acquati S., Adinolfi V., Di Bartolo P. (2022). Metabolic disorders and gastroenteropancreatic-neuroendocrine tumors (GEP-NENs): How do they influence each other? An Italian Association of Medical Oncology (AIOM)/Italian Association of Medical Diabetologists (AMD)/Italian Society of Endocrinology (SIE)/Italian Society of Pharmacology (SIF) multidisciplinary consensus position paper. Crit. Rev. Oncol. Hematol..

[22] Font-Burgada J., Sun B., Karin M. (2016). Obesity and Cancer: The Oil that Feeds the Flame. Cell Metab..

[23] Kompella P., Vasquez K.M. (2019). Obesity and cancer: A mechanistic overview of metabolic changes in obesity that impact genetic instability. Mol. Carcinog..

[24] Karczewski J., Begier B., Rafał K., Edyta S. (2019). Obesity and the Risk of Gastrointestinal Cancers. Dig. Dis. Sci..

[25] Uzunlulu M., Telci-Caklili O., Oguz A. (2016). Association between Metabolic Syndrome and Cancer. Ann. Nutr. Metab..

[26] Mallik R., Chowdhury T.A. (2018). Metformin in cancer. Diabetes Res. Clin. Pract..

[27] Gukovsky I., Li N., Todoric J., Gukovskaya A., Karin M. (2013). Inflammation, autophagy, and obesity: Common features in the pathogenesis of pancreatitis and pancreatic cancer. Gastroenterology.

[28] Byers T., Sedjo R.L. (2015). Body fatness as a cause of cancer: Epidemiologic clues to biologic mechanisms. Endocr.-Relat. Cancer.

[29] Duvillié B., Kourdoughli R., Druillennec S., Eychène A., Pouponnot C. (2020). Interplay Between Diabetes and Pancreatic Ductal Adenocarcinoma and Insulinoma: The Role of Aging, GeNENic Factors, and Obesity. Front. Endocrinol..

[30] Leoncini E., Carioli G., La Vecchia C., Boccia S., Rindi G. (2016). Risk factors for neuroendocrine neoplasms: A systematic review and meta-analysis. Ann. Oncol..

[31] Halfdanarson T.R., Bamlet W.R., McWilliams R.R., Hobday T.J., Burch P.A., Rabe K.G., Petersen G.M. (2014). Risk factors for pancreatic neuroendocrine tumors a clinic-based case-control study. Pancreas.

[32] Zhan H., Cong L., Zhao Y., Zhang T., Chen G. (2013). Risk factors for the occurrence of insulinoma: A case-control study. Hepatobiliary Pancreat. Dis. Int..

[33] Katz L.H., Levi Z., Twig G., Kark J.D., Leiba A., Derazne E., Liphshiz I., Keinan-Boker L., Eisenstein S., Afek A. (2018). Risk factors associated with gastroenteropancreatic neuroendocrine tumors in a cohort of 2.3 million Israeli adolescents. Int. J. Cancer.

[34] Feola T., Puliani G., Sesti F., Modica R., Centello R., Minotta R., Cannavale G., Di Meglio S., Di Vito V., Lauretta R. (2022). Risk factors for gastroenteropancreatic neuroendocrine neoplasms (GEP-NENs): A three-centric case-control study. J. Endocrinol. Investig..

[35] Santos A.P., Santos A.C., Castro C., Raposo L., Pereira S.S., Torres I., Henrique R., Cardoso H., Monteiro M.P. (2018). Visceral obesity and metabolic syndrome are associated with well-differentiated gastroenteropancreatic neuroendocrine tumors. Cancers.

[36] Santos A.P., Castro C., Antunes L., Henrique R., Cardoso M.H., Monteiro M.P. (2019). Disseminated Well-Differentiated Gastro-Entero-Pancreatic Tumors Are Associated with Metabolic Syndrome. J. Clin. Med..

[37] Oweira H., Mazotta A., Mehrabi A., Reissfelder C., Rahbari N., Betzler A., Elhadedy H., Soubrane O., Chaouch M.A. (2022). Using a Reinforced Stapler Decreases the Incidence of Postoperative Pancreatic Fistula After Distal Pancreatectomy: A Systematic Review and Meta-Analysis. World J. Surg..

[38] Gavin K.M. (2022). Sex Differences in Adipose Tissue Function. Endocrinol. Metab. Clin. N. Am..

[39] Wu H.Y., Li N.S., Song Y.L., Bai C.M., Wang Q., Zhao Y.P., Xiao Y., Yu S., Li M., Chen Y.J. (2020). Plasma levels of acylated ghrelin in patients with insulinoma and expression of ghrelin and its receptor in insulinomas. Endocrine.

[40] Barrea L., Muscogiuri G., Modica R., Altieri B., Pugliese G., Minotta R., Faggiano A., Colao A., Savastano S. (2021). Cardio-Metabolic Indices and Metabolic Syndrome as Predictors of Clinical Severity of Gastroenteropancreatic Neuroendocrine Tumors. Front. Endocrinol..

[41] Barrea L., Muscogiuri G., Pugliese G., Modica R., Laudisio D., Aprano S., Faggiano A., Colao A., Savastano S. (2021). Chronotype: What role in the context of gastroenteropancreatic neuroendocrine tumors?. J. Transl. Med..

[42] Stolzenberg-Solomon R.Z., Newton C.C., Silverman D.T., Pollak M., Nogueira L.M., Weinstein S.J., Albanes D., Männistö S., Jacobs E.J. (2015). Circulating leptin and risk of pancreatic cancer: A pooled analysis from 3 cohorts. Am. J. Epidemiol..

[43] Yuan Q.H., Zhang L.L., Xu Y., Chen X., Zhang B., Li L.X., Li S., Shang D. (2021). Circulating leptin and adiponectin levels in patients with pancreatic cancer. Chin. Med. J..

[44] Kim H.I., Lim H., Moon A. (2018). Sex differences in cancer: Epidemiology, genetics, and therapy. Biomol. Ther..

[45] Tevfik-Dorak M., Karpuzoglu E. (2012). Gender differences in cancer susceptibility: An inadequately addressed issue. Front. Genet..

[46] Lopes-Ramos C.M., Quackenbush J., DeMeo D.L. (2020). Genome-Wide Sex and Gender Differences in Cancer. Front. Oncol..

