# Supplementary material for: Selected Serum Biomarkers (Leptin, Chromogranin A, CA19-9, CEA) in Patients with Pancreatic Neuroendocrine Neoplasm and Associations with Metabolic Syndrome
Source: Cancers (Basel). 2023 Apr 18;15(8):2348. doi: 10.3390/cancers15082348 (PMC10137194; doi:10.3390/cancers15082348)
Supplement: Supplementary file 1 [file cancers-15-02348-s001.zip › cancers-2262560-supplementary.pdf]

cancers

Selected Serum Biomarkers (Leptin, Chromogranin A, CA19-9, CEA) in Patients with Pancreatic Neuroendocrine Neoplasm and Associations with Metabolic Syndrome

Table S1. Diabetes Federation diagnostic criteria of metabolic syndrome.

Title 1	IDF (2005)
Obligatory	Waist Circumference ≥ 94 cm in men or ≥ 80 cm in women
Number of abnormalities	≥ 2 of the following:
Triglycerides (mg/dl)	≥150
Cholesterol HDL (mg/dl)	< 40 in men or < 50 in women
Hypertension (mmHg)	≥ 130/85
Glucose	≥ 100

IDF, International Diabetes Federation; HDL, high-density lipoprotein.

Table S2. The comparison of the studied circulating markers in patients with pancreatic neuroendocrine tumors and controls.

Title 1	PanNEN; n = 106 (mean ± SD; median)	Controls; n = 40 (mean ± SD; median)	P
Leptin (ng/ml)	12.75 ± 14.48; 7.89	10.50 ± 7.82; 7.96	NS
CA 19-9 (U/ml)	12.89 ± 14.66; 9.43	5.60 ± 4.46; 4.10	<0.001
CEA (µg/l)	1.64 ± 1.60; 1.15	1.03 ± 0.68; 0.79	NS
CgA (ug/l)	173.39 ± 434.00; 48.13	75.35 ± 32.93; 77.50	NS

PanNEN, pancreatic neuroendocrine neoplasms; SD. standard deviation; NS, not significant; CA 19-9, carbohydrate antigen 19-9; CEA, carcinoembryonic antigen; CgA, chromogranin A. The analysis was performed with the Mann-Whitney U-test.

Table S3. The selected serum markers metrics in the detection of pancreatic neuroendocrine tumors.

Variable	AUC (95% CI)	SE	z score	p	Youden index %	cut-off value	Sensitivity %	Specificity %	Accuracy %
Leptin (ng/ml)	0.40 (0.39 - 0.59)	0.05	-0.25	NS	16	19.51	21	95	41
**CA 19-9 (U/ml)**	**0.73 (0.64 - 0.82)**	**0.04**	**5.12**	**<0.001**	**38**	**4.28**	**80**	**58**	**74**
CEA (µg/l)	0.61 (0.51 - 0.71)	0.05	2.24	NS	23	1.95	33	76	55
CgA (ug/l)	0.39 (0.30 - 0.48)	0.04	-2.47	NS	19	127.6	12	74	43

AUC, area under the curve; CI, confidential interval; SE, standard error; CEA, carcinoembryonic antigen; CA 19-9,

carbohydrate antigens CA 19-9; CgA, chromogranin A NS, not significant.

Table S4. The selected serum markers metrics in the differentiation of pancreatic neuroendocrine tumors according to BMI.

**Variable**	**AUC (95% CI)**	**SE**	**z score**	**p**	**Youden index J %**	**cut-off value**	**Sensitivity %**	**Specificity %**	**Accuracy %**
Weight (kg)	0.90 (0.84-0.96)	0.03	13.29	<0.001	65	67	95	71	83
Height (cm)	0.53 (0.42-0.64)	0.06	0.54	NS	11	176	33	78	55
Age (years)	0.62 (0.52-0.73)	0.05	2.25	NS	23	55.02	60	63	61
Glucose (mg/dl)	0.61 (0.51-0.72)	0.06	2.07	NS	20	90.50	69	51	60
TGs (mg/dl)	0.61 (0.50-0.72)	0.06	2.03	NS	23	72	86	37	62
Cholesterol (mg/dl)	0.57 (0.46-0.68)	0.06	1.29	NS	18	170	75	43	59
**Leptin (ng/ml)**	**0.71 (0.61-0.80)**	**0.05**	**4.11**	**<0.001**	**33**	**6.59**	**80**	**53**	**67**
CA 19-9 (U/ml)	0.56 (0.45-0.67)	0.06	1.10	NS	17	9.67	58	59	59
CEA (μg/l)	0.53 (0.41-0.64)	0.06	0.44	NS	16	0.81	71	45	59
CgA (ug/l)	0.44 (0.33-0.55)	0.06	-1.04	NS	5	32.88	67	37	53

AUC, area under the curve; CI, confidence interval, SE, standard error; NS, not significant. TGs, triglycerides; CA 19-9, carbohydrate antigen 19-9; CEA, carcinoembryonic antigen; CgA, chromogranin A; NS, not significant.

Table S5. The comparison of anthropometric and biochemical parameters depending on the presence/absence of the metabolic syndrome in patients with pancreatic neuroendocrine tumors.

	**Patients with MS,** *n* = 27 (mean ± SD; median)	**Patients without MS,** n=79 (mean ± SD; median)	p
Age (years)	61.19 ± 8.96; 58.52	49.73 ± 13.82; 50.37	< 0.001
Weight (kg)	80.42 ± 15.00; 82.90	68.68 ± 13.35; 68.00	< 0.01
Height (cm)	168.70 ± 8.22; 168.00	169.33 ± 9.79; 168.00	NS
BMI (kg/m2)	28.20 ± 4.67; 27.51	23.86 ± 3.60; 24.02	< 0.001

Cholesterol (mg/dL)	181.33 ± 55.43; 180.00	192.05 ± 47.19; 192.00	NS
TGs (mg/dL)	131.96 ± 53.80; 114.00	101.04 ± 60.04; 84.00	< 0.01
Glucose (mg/dL)	107.73 ± 30.90; 105.00	90.78 ± 18.54; 90.60	< 0.001
Leptin (ng/ml)	12.92 ± 9.13; 7.73	12.70 ± 15.95; 7.94	NS
CA 19-9 (U/ml)	14.81 ± 16.66; 13.63	12.24 ± 13.96; 8.90	NS
CEA (μg/l)	2.07 ± 1.81; 1.75	1.50 ± 1.50; 1.06	NS
CgA (ug/l)	220.87 ± 505.97; 63.34	157.16 ± 408.87; 42.22	NS

BMI, body mass index; SD. standard deviation; TGs, triglycerides; CA 19-9, carbohydrate antigen 19-9; CEA, carcinoembryonic antigen; CgA, chromogranin A; NS, not significant.

Table S6. The selected serum markers metrics in the differentiation of pancreatic neuroendocrine tumors depend on the presence/absence of the metabolic syndrome.

**Variable**	**AUC (95% CI)**	**SE**	**z score**	**p**	**Youden index J %**	**cut-off value**	**Sensitivity %**	**Specificity %**	**Accuracy %**
BMI (kg/m2)	0.78 (0.68 - 0.88)	0.05	5.35	< 0.001	47	24.8	89	58	66
Weight (kg)	0.72 (0.61 - 0.83)	0.06	3.86	< 0.001	37	82.90	52	85	76
Age (years)	0.75 (0.65 - 0.85)	0.05	5.08	< 0.001	44	55.02	82	62	67
Glucose (mg/dl)	0.73 (0.61 - 0.85)	0.06	3.69	< 0.001	50	101.20	70	80	77
TGs (mg/dl)	0.69 (0.58 - 0.81)	0.06	3.31	< 0.001	35	143	48	87	77
CEA (μg/l)	0.63 (0.51 - 0.75)	0.06	2.10	NS	30	1.71	56	75	70
CA 19-9 (U/ml)	0.58 (0.45 - 0.71)	0.07	1.20	NS	24	11.74	59	65	63
Leptin (ng/ml)	0.58 (0.46 - 0.69)	0.06	1.27	NS	23	4.62	89	34	48
CgA (ug/l)	0.56 (0.44 - 0.69)	0.06	1.00	NS	18	63.34	52	66	62
Height (cm)	0.48 (0.36 - 0.60)	0.06	-0.30	NS	7	167	63	44	49
Cholesterol (mg/dL)	0.45 (0.32 - 0.59)	0.07	-0.70	NS	6	173	67	39	46

AUC, area under the curve; CI, confidence interval, SE, standard error; NS, not significant. TGs, triglycerides; CA 19-9, carbohydrate antigen 19-9; CEA, carcinoembryonic antigen; CgA, chromogranin A; NS, not significant.

Table S7. The comparison of anthropometric and biochemical parameters depending on the sex in patients with pancreatic neuroendocrine tumors.

	Male; n=47 (mean ± SD; median)	Female; n=59 (mean ± SD; median)	P
Age (years)	(52,02±14,04; 52,73)	(53,15±13,47; 55,16)	NS
Weight (kg)	(80,92±11,87; 81,50)	(64,31±12,33; 66,00)	< 0.001
Height (cm)	(176,11±7,72; 176,00)	(163,64±6,47; 164,00)	< 0.001
BMI (kg/m2)	(26,17±4,07; 26,15)	(24,00±4,30; 23,95)	< 0.01
Cholesterol (mg/dl)	(178,70±41,61; 178,00)	(197,78±53,62; 194,00)	NS
TGs (mg/dl)	(121,36±69,96; 105,00)	(99,00±48,69; 89,00)	NS
Glucose (mg/dl)	(98,06±28,53; 93,70)	(92,74±18,21; 92,70)	NS
**Leptin (ng/ml)**	**(9,47±14,59; 6,92)**	**(15,37±13,97; 9,89)**	**0.01**
CA 19-9 (U/ml)	10,81±10,06; 8,41)	(14,56±17,39; 10,62)	NS
CEA (µg/l)	1,39±1,15; 1,11)	(1,85±1,86; 1,21)	NS
CgA (ug/l)	(81,90±117,36; 40,98)	(246,27±563,81; 53,09)	NS

BMI, body mass index; SD. standard deviation; TGs, triglycerides; CA 19-9, carbohydrate antigen 19-9; CEA, carcinoembryonic antigen; CgA, chromogranin A; NS, not significant.

Table S8. The selected serum markers metrics in the differentiation of pancreatic neuroendocrine tumors depending on the sex.

Variable	AUC (95% CI)	SE	z score	p	Youden index %	cut-off value	Sensitivity %	Specificity %	Accuracy %
Weight (kg)	0.16 (0.08-0.24)	0.04	-8.58	< 0.001	2	112	2	100	45
Height (cm)	0.10 (0.04-0.17)	0.03	-12.86	< 0.001	NA	NA	NA	NA	NA
Age (years)	0.52 (0.41-0.64)	0.06	0.41	NS	13	43	75	38	59
Glucose (mg/dl)	0.48 (0.37-0.59)	0.06	-0.31	NS	3	75	90	13	56
TGs (mg/dl)	0.40 (0.29-	0.06	-1.78	NS	NA	NA	NA	NA	NA

	0.51)									
Cholesterol (mg/dl)	0.61 0.72)	(0.50-	0.06	2.01	NS	22	212	39	83	59
**Leptin (ng/ml)**	**0.64 0.75)**	**(0.54-**	**0.05**	**2.65**	**< 0.01**	**30**	**11.09**	**49**	**81**	**63**
CA 19-9 (U/ml)	0.57 0.68)	(0.46-	0.06	1.84	NS	18	18.50	29	89	56
CEA (μg/l)	0.56 0.67)	(0.45-	0.06	0.99	NS	21	2.02	36	85	58
CgA (ug/l)	0.56 0.67)	(0.45-	0.06	1.14	NS	14	89.15	36	79	55

AUC, area under the curve; CI, confidence interval, SE, standard error; NS, not significant. TGs, triglycerides; CA 19-9, carbohydrate antigen 19-9; CEA, carcinoembryonic antigen; CgA, chromogranin A; NS, not significant.
